# Dataset about Mexico's forest diversity: Site locations of tree species, wood densities, and geographic database of forest-vegetation provinces

**DOI:** 10.1016/j.dib.2024.110186

**Published:** 2024-02-13

**Authors:** Martin Ricker, Miguel Á. Castillo-Santiago, Genaro Gutiérrez-García, Esteban M. Martínez-Salas, Edith Mondragón

**Affiliations:** a Departamento de Botánica, Instituto de Biología, Universidad Nacional Autónoma de México (UNAM), Ciudad Universitaria, Apartado Postal 70-233, Alcaldía Coyoacán, Ciudad de México 04510, Mexico; b Departamento de Observación y Estudio de la Tierra, la Atmósfera y el Océano; El Colegio de la Frontera Sur (ECOSUR), Carretera panamericana y Periférico sur s/n, Barrio María Auxiliadora, San Cristóbal de las Casas, Chiapas 29290, Mexico

**Keywords:** Basic wood-density, Floristic provinces, Major forest-vegetation types, Shapefile, Herbarium collections

## Abstract

A dataset about three topics is provided, as a follow-up to the article “Mexico's forest diversity: common tree species and proposed forest-vegetation provinces” by Ricker et al. [1]. Firstly, 6927 site locations are provided for 22,532 trees of 1452 species. Secondly, measurements of basic wood-densities are reported for 779 tree species, obtained from 5256 trunk-core samples from Mexico's national forest inventory, and ranging from 0.05 to 0.93 g/cm^3^. Third, the data and maps of the forest-vegetation provinces from [1] were updated with the new cartography of Mexico's vegetation and land use (base year 2018). The maps are available now in an adjusted presentation as a shapefile-set for ArcGIS, as well as map-package and image files.

Specifications Table

**Table:** 

Subject	Biological Sciences
Specific subject area	Distribution of tree species native to Mexico, wood densities of trees, delimitation of forest-vegetation provinces in Mexico
Data format	Raw Analyzed
Type of data	Table Map
Data collection	Herbarium specimens of trees were collected in the field across Mexico. Field teams of Mexico's national forest inventory visited 6927 sites between 2009 and 2017 (mainly between 2013 and 2015), taking herbarium specimens of 22,532 trees and core samples with a Pressler increment borer from 5256 tree trunks, representing 779 species. Details about measuring and calculating basic wood-density in the laboratory are described below. The geographic databases for the elaboration of maps were from Mexico's National Institute of Statistics and Geography (abbreviated INEGI in Spanish) and Mexico's National Commission for the Knowledge and Use of Biodiversity (abbreviated CONABIO in Spanish).
Data source location	Continental Mexico, with 6927 sample sites across all 32 states.
Data accessibility	Repository name: Mendeley Data; Data identification number (DOI) and direct URL to data: (a) https://doi.org/10.17632/77w6mfzwzj.3[2]: Excel file with 6927 site locations for 22,532 trees, representing 1452 species); (b) https://doi.org/10.17632/5d78g6mmng.3[3]: Excel file with basic wood-density of 5256 core-samples, representing 779 tree species); (c) https://doi.org/10.17632/sg6x6d5f84.3[4]: Figs. 2, 3a, and b as ArcGIS shapefile-set of 8 files, 3 ArcGIS map-package (mpk) or project files, or as three image (tif) files, and an Excel file with tabulated information).
Related research article	Ricker, M., J. Calónico, M.Á. Castillo-Santiago, A. Galicia, C. Kleinn, E.M. Martínez-Salas, E. Mondragón, M.A. Mora, L.J. Ramos, C.H. Ramos & S.A. Villela. 2022. Mexico's forest diversity: common tree species and proposed forest-vegetation provinces. *Forests* 13(1598): 1-31. https://doi.org/10.3390/f13101598. [1]

## 1 Value of the Data

• The study of wood densities was not included in [1]. The data is valuable for wood-property analyses, as wood density is related to other technical properties [5,6], and is necessary to estimate carbon storage in biomass for climate-change research [7].
• The list of 22,532 trees, representing 1452 native species, with geographic coordinates of their 6927 site locations (each one a site of 1600 m^2^) is valuable for analyzing and mapping the distributions of Mexico's tree species. Whereas a list with information about each tree species was provided as supplementary material for [1], the raw data with the information for each tree was not included. Such information is needed for the determination of tree conservation areas, analyzed for example in [8]. It is also essential input for descriptions of tree species with their distributions, such as in [9].
• Forest-vegetation provinces were proposed in maps, and statistically analyzed in [1], but the files of the maps for geographic information systems were not included. Furthermore, Mexico's Instituto Nacional de Estadística y Geografía (INEGI) has updated its map of land use and vegetation (from base year 2014 to 2018). Classification of vegetation has a long history in general, with specific challenges [10]. There are also potential applications, such as the large-scale territorial planning for managing and harvesting valuable species that are characteristic in their natural environment. Consequently, in [11] sustainable forest management was analyzed separately for 14 classes of European forest types. The included files are valuable for follow-up studies about the proposed forest-vegetation provinces, to verify their floristic distinction, and to refine their delimitations.

## 2 Background

The dataset represents a follow-up to a published article [1], where the objectives were to analyze floristically and statistically the data about trees from Mexico's national forest inventory, and to define forest-vegetation provinces. It would be desirable that other scientists continue to work with our data; therefore, we want to make it as accessible as possible. In addition to providing the data for each individual tree and the ArcGIS files of the (updated) maps, the wood-density data measured by us for 23% of the trees has never been published.

## 3 Data Description

### 3.1 Site locations of analyzed trees

In the Excel file “Mexico's forest diversity - Site locations for trees 26 Jan 2024.xlsx” [2], information is provided for 22,532 trees of 1452 species on 6927 sites, identified with herbarium specimens (though about three fourths without flowers or fruits). The original list of species identification for each tree that we used in [1], but did not published, was revised once more. As a result, 0.6% (127 of 22,659) tree entries were eliminated, because we considered their identification doubtful. Furthermore, a recent taxonomic revision for the Sapotaceae [12] was taken into account, leading to the change of two genus names. The file includes information for 17 variables that are explained in Table 1. Furthermore, the data of each Excel column is statistically summarized in the last table column.

**Table 1:** Variables in the Excel file “Mexico's forest diversity - Site locations for trees 26 Jan 2024.xlsx” [2], with information for 22,532 trees.

Nr	Variable	Explanations	Summary information
1	Collection code	Standardized code for the data base, consisting of the name of the company that was hired for the field work, the year of the inventory, and a collection code with the initials of the collector's name and his or her collection number.	Of the total (22,532 trees), 49.2% are from the field-work company INYDES, 38.4% from DIAAPROY, and 12.4% from AMAREF; 40.9% are from 2013, 36.2% from 2015, 16.9% from 2014, 3.1% from previous special collections, and 3.0% from 2016.
2	Species	The tree species were identified with (mostly sterile) herbarium specimens at the National Herbarium of Mexico (MEXU) at the Instituto de Biología (UNAM) in Mexico City.	The total is 1452 species; the mean is 15.5 trees per species, but the median is 3; the most frequent species are *Pinus leiophylla* (454 trees), *Quercus magnoliifolia* (390), and *Juniperus deppeana* (371).
3	Author	The abbreviated author(s) of the species name (see www.tropicos.org for complete information).	There are 746 different authors or author combinations.
4	Genus	The corresponding genus of the plant species.	The trees are from 472 different genera, the most frequent being *Quercus* (22.3% of the trees), *Pinus* (13.5%), and *Lysiloma* (3.7%).
5	Family	The corresponding family of the plant species.	The trees are from 117 different families, the most frequent being Fagaceae (22.3% of the trees), Fabaceae (17.3%), and Pinaceae (13.8%).
6	UPMID site code	Primary sampling-unit identifier (“Unidad Primaria de Muestreo - Identificador”), used by Mexico's Comisión Nacional Forestal (CONAFOR) for site identification since 2015; the 33,745 numbers on a national grid range from 1 to 311,922.	The trees were found on 6927 sites, with 1 to 33 trees from the same site; the mean is 3.3 trees per site and the median is 2.
7	Site's latitude (°)	The latitude of the center of the previously defined and established 1600 m^2^ field site, according to the World Geodetic System 1984 (WGS84), provided by the CONAFOR.	The 6445 distinct latitudes range from 14.5389° to 32.5064°.
8	Site's longitude (°)	The longitude of the center of the previously defined and established 1600 m^2^ field site, according to the World Geodetic System 1984 (WGS84); provided by the CONAFOR.	The 6684 distinct longitudes range from −116.6330° to −86.7669°.
9	Elevation (m)	Elevation in meters above sea level, derived from the geographic coordinates, according to the INEGI's model CEM 3.0 (“Continuo de Elevaciones Mexicano”), with raster resolution of 120 m.	The 2463 elevations range from −13 to 3944 m, with a mean of 1298 m and a median of 1317 m.
10	State	State, derived from the geographic coordinates, according to the INEGI.	The trees were in all 32 Mexican states; 19.1% of the trees were in Oaxaca, 12.2% in Durango, and 10.3% in Chihuahua.
11	Municipality	Municipality, derived from the geographic coordinates, according to the INEGI.	The trees were in 1336 municipalities, 54.0% of Mexico's total of 2475 municipalities (in 2023).
12	Major forest-vegetation type	In the Excel file in [4], the 183 categories of the land use and vegetation map (series VII) of the INEGI are grouped into seven major forest-vegetation types and “other surfaces”.	Most trees were in Coniferous forest (31.4%), followed by Highland broadleaf forest (21.7%), and Lowland dry forest (18.5%).
13	Floristic province	Rzedowski in [16] and Rzedowski & Reyna-Trujillo in [17] defined 15 mainland floristic provinces of Mexico.	Most trees were in the Western Mountain Range (23.7%), Pacific Coast (19.1%), and Southern Highlands (18.5%).
14	Collection date	Date when the herbarium specimens of the tree were collected in the field	The herbarium specimens of the trees were collected between March 2009 and October 2017, with a median date in July 2014.
15	Taxonomist who identified species	Herbarium specimens of the trees were identified at the National Herbarium of Mexico (MEXU) in the Instituto de Biología (UNAM) in Mexico City.	The herbarium specimens of the trees were identified by 64 taxonomists, but 90% were identified by only five taxonomists: Clara H. Ramos, Mauricio A. Mora, Jorge Calónico, Esteban M. Martínez, and Leandro J. Ramos.
16	Reported height (m)	Measured for smaller trees (< 8 m) with a flexometer, and for larger trees with an ultrasound hypsometer or a clinometer.	Heights ranged from 1 to 42 m, with a mean of 8.1 m and a median of 7.1 m; height data are missing for 7.4% of the trees.
17	Reported diameter (cm)	Diameter at breast height (dbh), measured with measuring tape.	Diameters ranged from 3 to 421 cm, with a mean of 19.2 cm and a median of 14.9 m; diameter data are missing for 7.4% of the trees.

### 3.2 Wood density

The diversity of wood densities has received increased intention in recent decades, in order to calculate carbon stocks in biomass for climate-change research [7]. However, data on wood densities is essential for various topics, from ecological and evolutionary analyses of trees and forests [13] to technological and economic analyses [5,6]. The Excel file “Mexico's forest diversity - Wood densities 25 Jan 2024.xlsx” [3] contains 22 variables that are explained in Table 2.

**Table 2:** Variables in the In the Excel file “Mexico's forest diversity - Wood densities 26 Jan 2024.xlsx” [3], with information for 5256 tree core-samples or 23.3% of the trees referred to in Table 1, and always 1 sample per tree.

Nr	Variable	Explanations	Summary information
1	Collection code	The collection codes represent a subset (23.3%) of those mentioned already in Table 1.	Of the total (5256 tree core-samples), 63.0% are from the field-work company DIAAPROY, 32.1% from INYDES, and 5.0% from AMAREF; 77.9% are from 2013, 14.4% from 2014, and 7.6% from 2015.
2	Species	The tree species were identified with (mostly sterile) herbarium specimens at the National Herbarium of Mexico (MEXU) at the Instituto de Biología (UNAM) in Mexico City.	The total is 779 species; the mean is 6.7 tree core-samples per species, but the median is 2; the most frequent species are *Pinus leiophylla* (177 samples), *Pinus oocarpa* (137), and *Pinus durangensis* (122); on the other hand, 319 species were represented by a single core sample, and 125 species by two.
3	Author	The abbreviated author(s) of the species name (see www.tropicos.org for complete author names).	There are 435 different authors or author combinations.
4	Genus	The corresponding genus of the plant species.	The core samples are from 312 different genera, the most frequent being *Pinus* (25.2% of the samples), *Quercus* (17.5%), and *Bursera* (4.9%).
5	Family	The corresponding family of the plant species.	The core samples are from 97 different families, the most frequent being Pinaceae (25.9% of the samples), Fagaceae (17.5%), and Fabaceae (10.1%).
6	Basic wood-density (g/cm^3^)	The oven-dry mass of the core sample, divided by its calculated green volume.	The 5256 density data range from 0.047 to 0.925 g/cm^3^, with a mean of 0.530 g/cm^3^ and a median of 0.521 g/cm^3^.
7	Core-sample length (cm)	The overall length of the cylinder-shaped samples.	The lengths range from 0.56 to 26.99 cm, with a mean of 6.45 cm and a median of 6.11 cm.
8	Elliptic- cylinder volume (cm^3^)	The employed Pressler borers extract core samples of 0.508 cm diameter; for improved volume calculation, two diameters perpendicular to each other were measured, allowing for a slightly elliptic shape of the bases in some cases.	The core-sample volumes range from 0.097 to 5.470 cm^3^, with a mean of 1.285 cm^3^ and a median of 1.218 cm^3^.
9	Wood density at ambient humidity (g/cm^3^)	The mass of the core sample, divided by its volume, at ambient humidity (in the herbarium) without drying or volume adjustment.	These density measurements range from 0.059 to 1.243 g/cm^3^, with a mean of 0.660 g/cm^3^ and a median of 0.641 cm^3^.
10	Eliminated moisture (%)	The mass of eliminated water in the oven, as a percentage of the subsequent dried mass.	The percentages range from 0% to 92.5%, with a mean of 9.0% and a median of 9.1%.
11	UPMID site code	Primary sampling-unit identifier (“Unidad Primaria de Muestreo - Identificador”), used by Mexico's Comisión Nacional Forestal (CONAFOR) for site identification since 2015; the 33,745 numbers on a national grid range from 1 to 311,922.	The core samples are from trees on 1916 sites, with 1 to 18 trees from the same site; the mean is 2.7 trees per site and the median is 2.
12	Site's latitude (°)	The latitude of the center of the previously defined and established 1,600 m^2^ field site, according to the World Geodetic System 1984 (WGS84), provided by the CONAFOR.	The 1877 distinct latitudes range from 15.2081° to 32.4572°.
13	Site's longitude (°)	The longitude of the center of the previously defined and established 1600 m^2^ field site, according to the World Geodetic System 1984 (WGS84); provided by the CONAFOR.	The 1894 distinct longitudes range from −116.1200° to −87.2789°.
14	Elevation (m)	Elevation in meters above sea level, derived from the geographic coordinates, according to the INEGI's model CEM 3.0 (“Continuo de Elevaciones Mexicano”), with raster resolution of 120 m.	The 1337 elevations range from −4 to 3834 m, with a mean of 1496 m and a median of 1580 m.
15	State	State, derived from the geographic coordinates, according to the INEGI.	The core samples are from trees in 31 of the 32 Mexican states (without samples from Colima); 31.3% of the trees were from Oaxaca, 14.3% from Guerrero, and 11.1% from Chihuahua.
16	Municipality	Municipality, derived from the geographic coordinates, according to the INEGI.	The core samples are from trees in 717 municipalities, 29.0% of Mexico's total of 2475 municipalities (in 2023).
17	Major forest-vegetation type	In the Excel file in [4], the 183 categories of the land use and vegetation map (series VII) of the INEGI are grouped into seven major forest-vegetation types and “other surfaces”.	Most core samples are from trees in Coniferous forest (41.1%), followed by Lowland dry forest (20.0%), and Lowland evergreen forest (11.4%).
18	Floristic province	Rzedowski in [16] and Rzedowski & Reyna-Trujillo in [17] defined 15 mainland floristic provinces of Mexico.	Most core samples are from trees in the Southern Highlands (26.0%), Western Mountain Range (21.7%), and Pacific Coast (18.3%).
19	Collection date	Date, when the herbarium specimens of the tree were collected in the field.	The core samples were collected between April 2013 and October 2015, with a median date in August 2013.
20	Taxonomist who identified species	Herbarium specimens of the trees were identified at the National Herbarium of Mexico (MEXU) in the Instituto de Biología (UNAM) in Mexico City.	The corresponding herbarium specimens of the trees were identified by 30 taxonomists, but 88% were identified by only five taxonomists: Jorge Calónico, Clara H. Ramos, Mauricio A. Mora, Esteban M. Martínez-Salas, and Rosalinda Medina.
21	Reported height (m)	Measured for smaller trees (< 8 m) with a flexometer, and for larger trees with an ultrasound hypsometer or a clinometer.	The core samples are from trees that ranged in height from 1.3 to 37 m, with a mean of 9.3 m and a median of 8.0 m; height data are missing for 4.2% of the trees.
22	Reported diameter (cm)	Diameter at breast height (dbh), measured with measuring tape.	The core samples are from trees that ranged in diameters from 3.9 to 237 cm, with a mean of 21.6 cm and a median of 17.1 m; diameter data are missing for 4.2% of the trees.

**Fig. 1:**
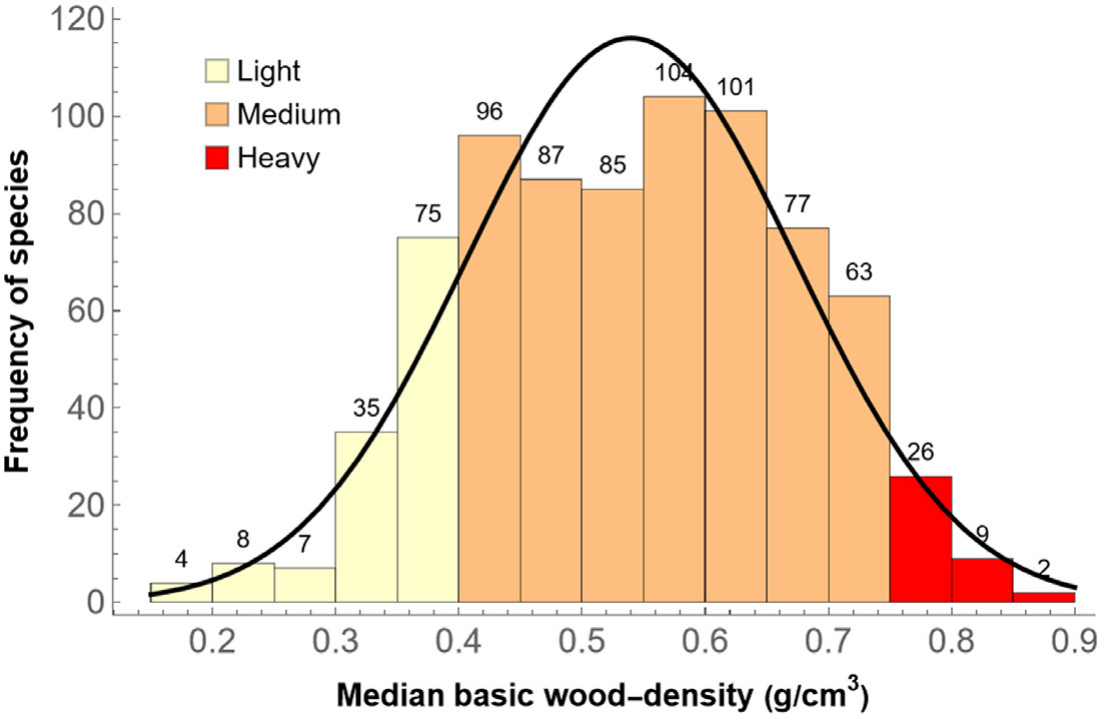
For 129 species, the wood can be considered light, for 613 species medium, and for 37 species heavy.

The histogram in Fig. 1 shows the statistical distribution of the median basic wood-density for the 779 species; the median was used, because the statistical distributions within species are sometimes highly asymmetrical (but note that 319 species were represented by only one core-sample, and 125 species by only two). On pages 322–323 in [14], three ranges of wood densities are distinguished: up to 0.4 g/cm^3^ is considered a low density (“light wood”), between 0.4 and 0.75 g/cm^3^ a medium density, and starting at 0.75 g/cm^3^ a high density (“heavy wood”). The histogram shows the three ranges in different colors: There are 129 species with low density (yellow), 613 species with medium density (orange), and 37 species with high density (red).

The three species with the lightest wood and at least three samples were *Erythrina americana* (0.197 g/cm^3^ median wood density), *Cochlospermum vitifolium* (0.207 g/cm^3^), and *Heliocarpus appendiculatus* (0.219 g/cm^3^). The three species with the heaviest wood and at least three samples were *Ebenopsis ebano* (0.866 g/cm^3^), *Acacia bilimekii* (0.822 g/cm^3^), and *Olneya tesota* (0.807 g/cm^3^). Mexico is home to some of the lightest woods in the world, such as *Ochroma pyramidale* (“balsa”), with wood densities as low as 0.06 g/cm^3^ [15], as well as the heaviest woods, such as *Krugiodendron ferreum* (“black ironwood”) with densities as high as roughly 1.3 g/cm^3^ (https://florida.plantatlas.usf.edu/plant.aspx?id=3386). Our *Ochroma pyramidale* sample measured 0.238 g/cm^3^, which falls into the range of 0.06–0.38 g/cm^3^ reported in [15], and unfortunately we had no sample of *Krugiodendron ferreum*.

### 3.3 Forest-vegetation provinces

**Fig. 2:**
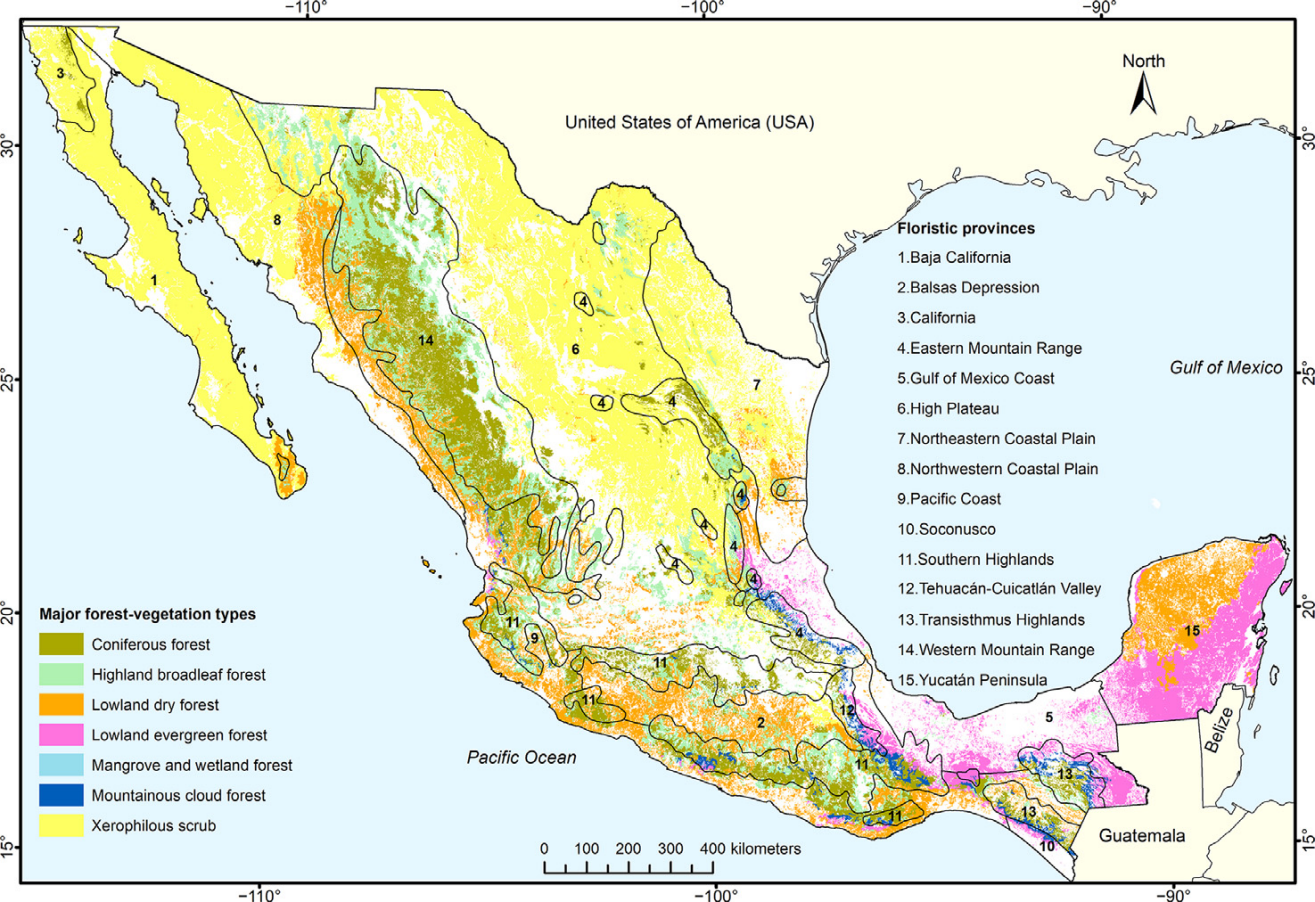
Mexico's forest-vegetation provinces are determined by combining two maps: The first one is Rzedowski's map of 15 floristic provinces for continental Mexico, listed on the right, and indicated with lines and numbers. The second one is our map of major forest-vegetation types, listed on the left, and indicated with colors. The map of major forest-vegetation types is based on the map of land use and vegetation from the INEGI. “Mangrove and wetland forest” is not included to determine forest-vegetation provinces.

The forest-vegetation provinces provide a framework for comparing and analyzing Mexico's forests, in terms of structure and floristic composition, and considering associated factors of climate and ecology. Their spatial distribution is the result of overlapping two maps: Rzedowski's map of 15 floristic provinces for continental Mexico [16,17], and our map of major forest-vegetation types, which in turn is based on the map of land use and vegetation from the INEGI (Fig. 2).

The forest-vegetation provinces were updated here, after updating the major forest-vegetation types, derived from the INEGI's cartography. The seven major forest-vegetation types correspond now to the base year 2018, instead of 2014 in [1]. The source for Mexico's floristic provinces according to Rzedowski remained unchanged for the combination of the two sources into the forest-vegetation provinces.

In [1], we did not provide the source files for the geographic information systems of our maps. This is done here, with the idea that scientists interested in doing follow-up work can use and refine the cartography of specific forest-vegetation provinces. There are three folders provided that represent Figs. 2, 3a, and 3b in different formats: a folder with eight files of an ArcGIS shapefile-set; another folder with three ArcGIS map-package (mpk) or project files; and a third folder with three image (tif) files. The mpk files contain the geographic data sets plus all necessary data to recreate a cartographic design, as in Fig. 2.

**Fig. 3a:**
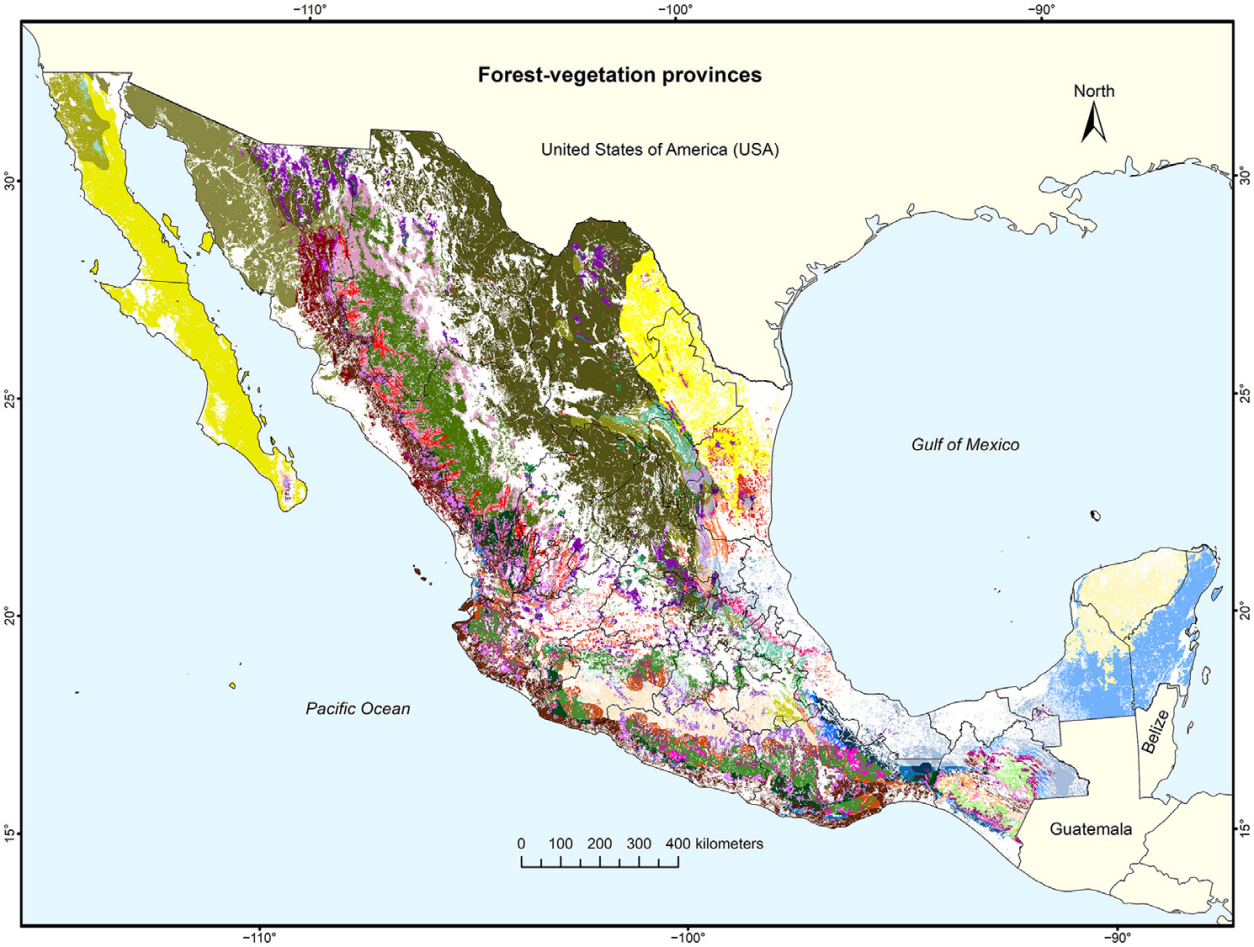
Mexico's 73 forest-vegetation provinces, ranging in size from 0.0004% to 14.2% of Mexico's continental area.

Furthermore, there is an Excel file with tabulated information on two spreadsheets (“Mexico's forest diversity - Forest-vegetation provinces 28 Dec 2023.xlsx”). The first spreadsheet (“Conversion to major types”) shows our conversion from the INEGI's categories of 183 land use and vegetation types into seven major forest-vegetation types plus “other surfaces”. The 183 terms are given in English and Spanish. The second spreadsheet (“Forest-vegetation provinces”) provides the names of all forest-vegetation provinces in English and Spanish. The names are combinations of the term for the major forest-vegetation type and after a hyphen the term for the floristic province. The subsequent columns provide for each forest-vegetation province the absolute area (in km^2^), relative area (in %), and their sums for each of the six major forest-vegetation types. “Mangrove and wetland forest” is excluded from the determination of forest-vegetation provinces, because salty or sweet water dominates its floristic composition.

The resulting map of the 73 forest-vegetation provinces is shown in Fig. 3a, with a different color for each province. The legend for the 73 colors is given in Fig. 3b. Whereas the map requires enlargement to appreciate the details of smaller forest-vegetation provinces, it transmits the floristic diversity of Mexico's forest vegetation.

**Fig. 3b:**
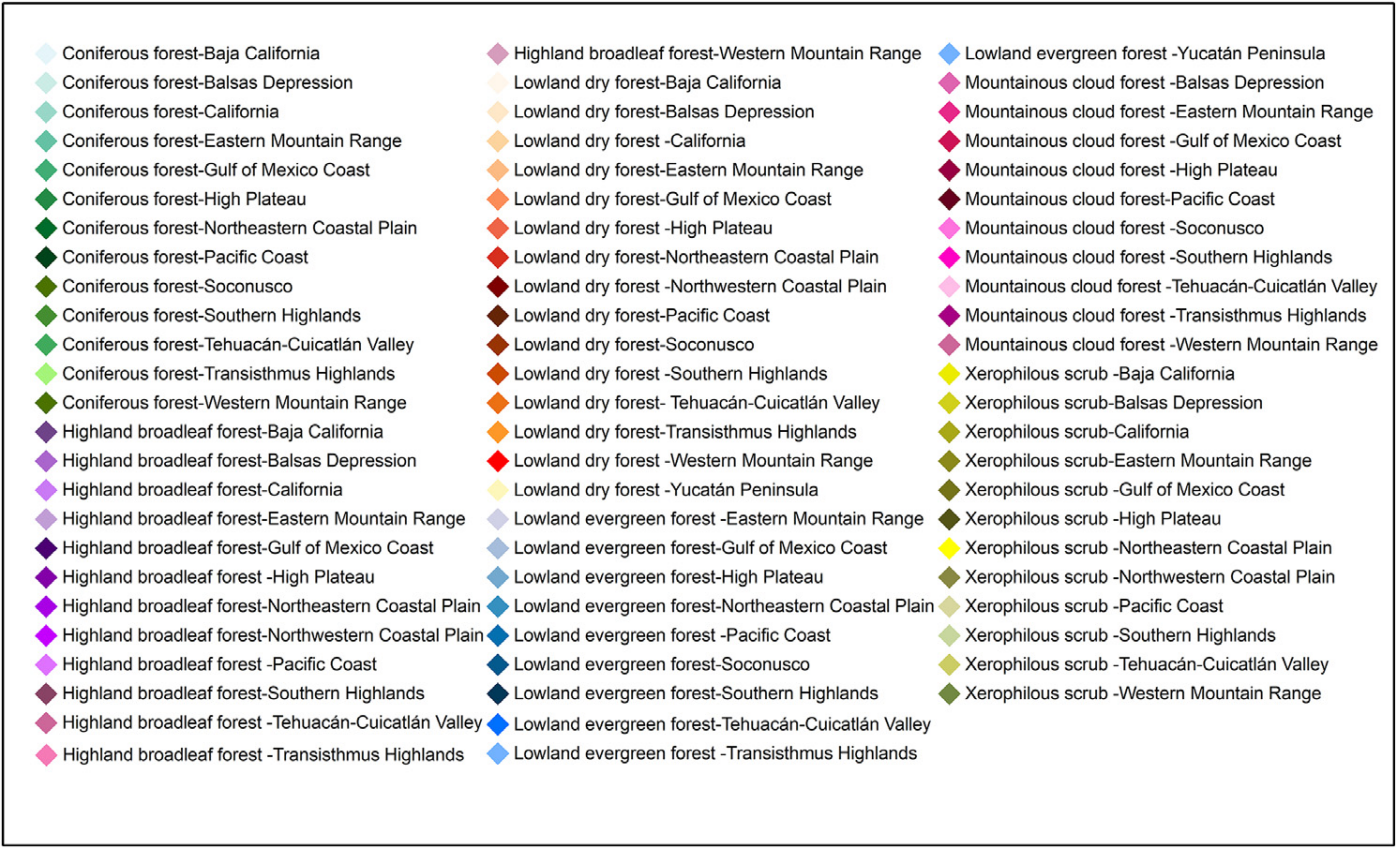
The legend for Fig. 3a, with the names of the 73 forest-vegtation provinces.

Comparing the updated data for the base year 2018 in the mentioned Excel file with the data in the supplementary material S2 in [1] for the base year 2014, there are now 73 instead of 75 forest-vegetation provinces. Two small provinces disappeared: “Mountainous cloud forest - Northwestern Coastal Plain” of 2 km^2^ and “Mountainous cloud forest - Yucatán Peninsula” of 6 km^2^. In the updated version here, the area of the forest-vegetation provinces ranges from 7 km^2^ (”Mountainous cloud forest - Western Mountain Range”) to 278,514 km^2^ (“Xerophilous scrub - High Plateau”). The mean area is 16,596 km^2^ and the median area 3208 km^2^. Whereas the largest forest-vegetation province covers 14.2% of Mexico´s continental area (1,954,658 km^2^ according to the CONABIO in November 2019), 36 provinces cover less than 0.1% each. The sum of the areas of all forest-vegetation provinces is 1,211,501 km^2^, or 62.0% of Mexico's continental land area, which is slightly higher than the area reported in [1] (60.42%).

## 4 Experimental Design, Materials and Methods

### 4.1 Site locations and information for trees

The field methods for taking herbarium specimens of trees and species identifications are described in [1], and in more detail in Spanish in [18]. Mexico's national forest inventory (Inventario Nacional Forestal y de Suelos) covers all of Mexico's continental area. Mexico's national forest agency CONAFOR (Comisión Nacional Forestal) hired companies that carried out the field work (here AMAREF, DIAAPROY, and INYDES). The companies hired people that form three-person field crews, generally including biologists, foresters, or related professionals. The goal was to make one collection of each distinct tree species per 1600 m^2^ plot, where a collection consists ideally of several “duplicate” herbarium specimens from the same tree. That goal was not always met, either because field crews did not recognize a given tree as a distinct species, or because of difficult access to the twigs with leaves and flowers or fruits of large trees. The dried herbarium specimens were sent or delivered to Mexico's National Herbarium (MEXU) at the Biology Institute, where they were deep-frozen (−70 °C) for three days to kill insects, before being processed further. In addition to collecting the herbarium specimens, tree and site information was measured (height, diameter, geographic coordinates) or recorded, and photos of each tree were taken by the field crews.

Plant families were identified and specimens separated accordingly, before proceeding with species identifications. Traditional species (or taxa) identification was carried out by specialists who already knew many of the diagnostic characters, but also compared identified specimens in the herbarium and consulted literature. Only trees that could be identified at the species level were included here.

### 4.2 Wood density measurements

The field methods for taking core samples from tree trunks with a Pressler increment borer are described in Spanish in [19]. Wood density varies highly with water content, because both mass and volume change with different moisture contents. Therefore it is necessary to standardize the density measurements. For technical purposes, wood density is often compared at 12% moisture. However, for biomass estimation *basic wood-density* is generally used, which is oven-dry mass at 0% moisture per green or water-saturated volume (in g/cm^3^). Here we measured and calculated basic wood-density. Wood density at 12% moisture and basic wood-density can be estimated from each other with a mathematical formula [20].

For calculating basic wood-density, the volume of the wood sample would ideally be measured immediately after collection from the tree, which is rarely practical. When the core sample dries, its volume shrinks up to 20% (page 379 in [21]). The shrinkage is usually 1.5 to 2.5 times greater in the tangential direction than in the radial direction (page 229 in [22]). To estimate the basic wood-density $\left(D_{b}\right)$ later in the laboratory, without having measured green volume, we used the following formula (whose origin is explained below):

(1) $$D_{b}=\frac{29466\cdot D_{w}}{30000+7950\cdot D_{w}+300\cdot w-265\cdot D_{w}\cdot w},$$

where $D_{w}$ is the wood density at water content w, in a range between 0% and 200% of its oven-dry mass. The maximum possible water content decreases with higher wood density, from above 250% to less than 100% (Fig. 2 in [23]). To be exact, the calculated density corresponds to a temperature of 20 °C, but temperature changes have minimal effect. In order to apply (1), the measurement procedure was the following:

(1) The two transversal circular areas on the extremes of the dry (under ambient humidity) core sample were honed down with sandpaper, to get perpendicular areas.
(2) The length of the core sample was measured (in cm) with a caliper, with a precision of 0.01 mm.
(3) The Pressler increment borer has an inner diameter of 0.200 inches (0.508 cm). After drying, the circular shape may have deformed slightly. To take into account a transversal shape like an ellipse, the minimum and maximum diameters $(d_{Min}$ and $d_{Max})$ of the transversal area were measured with a caliper (in mm), so that the volume formula was $volume=d_{Min}\cdot d_{Max}\cdot \pi \cdot l/400$ in cm^3^, where l is the length of the core sample, and π≈3.14159.
(4) The mass $m_{1}$ of the core sample was measured (in g) with the included water content, using an analytical scale with a precision of 0.001 g, and the wood density $D_{w}$ was calculated.
(5) The core samples were dried at 105 °C for 48 h in a gravity convection oven (Binder redLine RE-53). The mass $m_{2}$ was measured immediately after taking the core sample out of the oven. The relative water content of the core sample was calculated as $w=100\%\cdot \left(m_{1}-m_{2}\right)/m_{2}.$
(6) Having obtained $D_{w}$ and w for a given core sample, $D_{b}$ was determined with (1).

Next, we will explain how Eq. (1) was derived. Wood densities are often measured relative to the density of water, and the ratio is called “specific gravity” (page 322 in [14], [24]). The to-be-measured piece of wood is forced under water to measure the volume displacement (without air bubbles). Numerically, basic specific-gravity (without units) is almost equal to basic wood-density (in mass per volume), except that water slightly changes its density as a function of its temperature, with its highest density of 1 g/cm^3^ being at about 4 °C (and 1 atm pressure). The advantages of determining (basic) specific density in water are that irregularly shaped samples can be measured, and the green volume is again approximated when water absorption causes re-swelling of dry samples. For small samples, however, the procedure to force and measure accurately the wood under water can be difficult.

The following formula to estimate $D_{w}$ from the basic specific-gravity $G_{b}$ and w is given on page 3 in [23]:

(2) $$D_{w}=\left\{ \begin{array}{c} \frac{G_{b}\cdot \left(1+\frac{w}{100}\right)}{1-0.265\cdot \frac{30-w}{30}\cdot G_{b}}\;\text{for}\;0\%\leq w\leq 30\%,\\ G_{b}\cdot \left(1+\frac{w}{100}\right)\;\text{for}\;w>30\%. \end{array}\right.$$

The formula reflects the observation that wood dried below its fiber-saturation point undergoes shrinkage (roughly below 30% water content, but depending on the species). Stamm carried out regression analysis of external volume shrinkage as a function of basic specific-gravity (in the range of 0.25–0.65) for different species (pages 220–223 in [22]). He found a linear regression line for 52 softwood species with a slope of 0.26 and *Y*-intercept of 0, with a range of volume shrinkage from 6% to 16% for basic specific-gravity from 0.25 to 0.65. For 106 hardwood species, the slope was 0.27, with a range of volume shrinkage from 7% to 23% for basic specific-gravity from 0.3 to 0.9. The average of the two slopes is 0.265, and represents an empirical coefficient of proportional external volume shrinkage as a function of basic specific-gravity.

Here we want to determine basic wood-density $(D_{b}),$ rather than basic wood-gravity $(G_{b}),$ calculated as

(3) $$D_{b}=G_{b}\cdot \rho_{water}.$$

We will use a density of water at a temperature of 20 °C, being 0.9982 g/cm^3^. How sensitive is the basic wood-density to a change in temperature? For example, the density of water changes from 0.9982 at 20 °C to 0.9957 at 30 °C, a change of −0.0025%. The basic wood-density according to (3) changes by $\Delta D_{b}=G_{b}\cdot \left(-0.0025{g/\text{cm}}^{3}\right)$, which at $G_{b}=0.2$ results in a change of −0.0005g/cm^3^, and at $G_{b}=1$ in −0.0025g/cm^3^. This magnitude of variation is negligible for our purposes.

Using (2) for the wood density $D_{w},$ substituting $G_{b}=D_{b}/\rho_{water},$ and employing the variable name $s_{Reg}$ for the above average regression slope of 0.265 results in

$$D_{w}=\frac{3\cdot D_{b}\cdot \left(100+w\right)}{300\cdot \left(\rho_{water}-s_{Reg}\cdot D_{b}\right)+10\cdot s_{Reg}\cdot D_{b}\cdot w},$$

and solving for$D_{b}$ in

(4) $$D_{b}=\frac{300\cdot D_{w}\cdot \rho_{water}}{3\cdot w+300\cdot \left(1+s_{Reg}\cdot D_{w}\right)-10\cdot s_{Reg}\cdot D_{w}\cdot w},$$

where $\rho_{water}$ and $D_{b}$ must be measured in the same units of mass per volume (here g/cm^3^). Finally, using from above $s_{Reg}=0.265=265/1000$ and $\rho_{water}=0.9982=9982/10000$ g/cm^3^, expressed as ratios to calculate exact numbers, yields (1).

How sensitive is (4) to the average regression slope $s_{Reg}=$0.265 from [22]? For example, for $D_{w}=0.5$ g/cm^3^ and w=12%, $D_{b}=0.409$ g/cm^3^. Using (4) with a 20%-increase of 0.265 ($s_{Reg}=$ 0.318) causes $D_{b}$ to decrease by 0.3% at $D_{w}=0.1$ g/cm^3^, and by 3.6% at $D_{w}=1.6$ g/cm^3^ (the result does not depend on $\rho_{water}$). The inverse changes for a 20%-decrease of 0.265 are similar. Consequently, the result is not very sensitive to the exact numerical value of the above-average regression slope.

### 4.3 Creation of maps with forest-vegetation provinces for Mexico

The maps of Figs. 2 and 3 were created with the software ArcGIS 10.8.1, using the following input layers:

• Land use and vegetation map, series VII (base year 2018, presented in 2021) on a scale of 1:250,000, from the INEGI's portal at https://www.inegi.org.mx/app/biblioteca/ficha.html?upc=889463842781;
• Floristic provinces of Mexico, delimited in the map from Rzedowski & Reyna-Trujillo [17] on a scale of 1:8,000,000, from the CONABIO's portal at http://www.conabio.gob.mx/informacion/gis/ [Regionalización - Bióticas - Divisiones florísticas]; the province of Guadalupe island was not included here, because it is outside of continental Mexico;
• Political divisions of Mexico's states on a scale of 1:50,000, from the INEGI's portal at https://www.inegi.org.mx/temas/mg/ [Área geográfica Estados Unidos Mexicanos, Versión 2022, Capas AGEE].

The mentioned sites also include metadata and documentation for the maps. Whereas in [1] series VI (base year 2014) was used, here it is the new series VII (base year 2018). To create the new version, the INEGI used Landsat satellite images for photointerpretation and verification in the field. Series VII distinguishes 108 vegetation categories that the INEGI identified and classified according to physiognomy, deciduousness, climate, species dominance, and successional stage. In addition to vegetation categories, land use and cover - such as agriculture, water bodies, or urban areas - are classified, which elevates the number of categories to 183. In the Excel file (first sheet) of [4], the name of each category is given first in Spanish, then its translation to English, and in the third column our conversion to either one of seven major forest-vegetation types or “Other surfaces”.

The limits of floristic provinces from [17] are approximate. They are also on a 32-times larger scale than the major forest-vegetation types. Some limits of the floristic provinces were slightly modified here, to agree adequately with the major forest-vegetation types without creating artificial polygons. The ArcGIS tools “Dissolve” and “Intersect” were used.

The shapefile-set consists of seven necessary files for ArcGIS, plus a file with the extension “lyr” that provides optional editorial information (for example, about colors). All eight files of the set have the common name “Forest vegetation provinces”, but each has a distinct extension. The reference system has the following specifications (see the text file “Forest vegetation provinces.prj”):

• System of projected coordinates: Albers equal-area conic projection (GCS_GRS_1980_IUGG_1980);
• False easting: 2,500,000.0 m.
• False northing: 0.0 m.
• Central meridian: −102.0°.
• Standard parallel 1: 17.5°.
• Standard parallel 2: 29.5°.
• Latitude of origin: 12.0°.
• Linear unit: meter.
• Datum: GRS80.
• Base meridian: Greenwich.
• Angular unit: Degree.

Furthermore, the following seven attributes are used, when the shapefile-set is opened in ArcGIS:

• clvePF: integer number as a code for the forest-vegetation province.
• cve_Prov: integer number as a code for the florístic province.
• cve_VT: integer number as a code for the major forest-vegetation type.
• Forest_VT: text for the major forest-vegetation type.
• km^2^: real number for the area in km^2^^.^
• Prov: text for the florístic province.
• ProvFore: text for the forest-vegetation province.

## Limitations

Concerning the taxonomic identifications of the tree species, the usual limitations exist in biologically diverse countries like Mexico, where there are approximately 3000 tree species (page 2 in [1]). New species are still being described, taxonomic changes are common, and a lack of taxonomic specialists for all plant families is a problem. Whereas any taxonomist can commit errors, the advantage of determining here a large number of herbarium specimens of the trees by a small group of experts is the consistency of the identifications (five taxonomists identified 90% of the 22,532 specimen collections).

Wood densities are variable from inner to outer wood, and to a much lesser extent among trunk heights [25,26], as well as in response to trunk inclination and environmental influences [27] and genetic differences within species [28]. One core sample per tree, and especially a short core sample, indicates only one value from the overall within-tree range of wood density. It was not possible here to distinguish heartwood from sapwood, or early from late wood. Thus, the variations within and among trees are not distinguishable. Nevertheless, in an assessment of 460 species with at least two trees, 69% of the variance of wood density was found among species, and 31% within species (among samples).

The delimitations of forest-vegetation provinces should be considered a first approximation for further research. Whereas the underlying floristic provinces were proposed and delimited by the highly regarded Mexican botanist Jerzy Rzedowski [16,17], that work was carried out over three decades ago, without much subsequent research for verification and refinement. On the other hand, the cartography of the INEGI about land use and vegetation is updated every few years (now being in its seventh version).

## Ethics Statement

These data are primary data and do not include any human subjects, animal experiment, or social media platforms.

## CRediT authorship contribution statement

**Martin Ricker:** Conceptualization, Methodology, Formal analysis, Writing – original draft, Writing – review & editing, Visualization, Investigation, Project administration, Funding acquisition. **Miguel Á. Castillo-Santiago:** Methodology, Writing – original draft, Visualization, Supervision. **Genaro Gutiérrez-García:** Methodology, Investigation. **Esteban M. Martínez-Salas:** Investigation, Validation. **Edith Mondragón:** Methodology, Formal analysis, Visualization.

## Data Availability

Mexico's forest diversity: site locations for trees (Original data) (Mendeley Data). Mexico's forest diversity: forest-vegetation provinces (Original data) (Mendeley Data). Mexico's forest diversity: wood densities (Original data) (Mendeley Data).
